# Automated Directional Measurement System for the Acquisition of Thermal Radiative Measurements of Vegetative Canopies

**DOI:** 10.3390/s90301409

**Published:** 2009-03-03

**Authors:** Joris Timmermans, A.S.M. Ambro Gieske, Christiaan van der Tol, Wout Verhoef, Zhongbo Su

**Affiliations:** 1 International Institute for Geo-Information Sciences and Earth Observation (ITC) / Hengelosestr. 99, P.O. Box 6, 7500 AA Enschede, the Netherlands; E-Mails: Gieske@itc.nl (A.G.); tol@itc.nl (C.T.); verhoef@itc.nl (W.V.); b_su@itc.nl (Z.S.); 2 National Aerospace Laboratory (NLR) / NLR Amsterdam, Anthony Fokkerweg 2, 1059 CM, Amsterdam, the Netherlands.

**Keywords:** Thermal, Directional, Brightness, Temperatures, Goniometer

## Abstract

The potential for directional optical and thermal imagery is very large. Field measurements have been performed with a goniometer on which thermal instruments were attached. In order to reduce dynamical effects the goniometer was adjusted to run in automated mode, for zenith and azimuthal direction. Directional measurements were performed over various crops with increasing heterogeneity. The improvements to the goniometer proved successful. For all the crops, except the vineyard, the acquisition of the directional thermal brightness temperatures of the crops went successfully. The large scale heterogeneity of the vineyard proved to be larger then the goniometer was capable of. The potential of directional thermal brightness temperatures has been proven.

## 1. Introduction

Passive remote sensing has become a necessary tool for monitoring large scale processes. Remote sensing in the optical and thermal domain has been used to retrieve surface parameters such as thermal emissivity [1], leaf area index [2] and to map evapotranspiration [3].

The accuracy in retrieved surface parameters is influenced by level of homogeneity of canopy and the pixel size of the images. Aggregation of the reflected/emitted radiation over large surfaces results in large errors for heterogeneous canopies [4–5]. For example, the ASTER sensor has a nadir looking resolution of about 90 meters in the thermal spectrum [6]. The radiation emitted by the sub-pixel processes are then averaged to a singular value per pixel. This makes it impossible to understand these subpixel features if only images of one viewing angle are used.

Directional remote sensing has the potential to produce higher accuracy retrieval of surface parameters than nadir-only remote sensing [7–8]. Reduction of signal to noise ratios (SNR) can be achieved by averaging multiple images and differences in measured spectra for different viewing angles can be exploited [9]. It was shown by [10,11] that radiation reflected by a sparse canopy varies a great deal between oblique and nadir viewing angles. They were able to take advantage of these directional variations to retrieve with better precision the leaf area index.

The use of optical directional imagery requires the knowledge of reflectance factors like the hemispherical-directional reflectance factor (HDRF) and the Bi-directional reflectance factor (BRDF). Analogous to presented research it was shown [12] the requirement of knowledge on thermal directional signatures for thermal directional images. These directional signatures can be simulated using radiative transfer models like SAIL [13] and DART [14] or must be measured on ground.

The directional viewing of the ground can be achieved using a goniometer [15,16]. Sensors like field spectrometers, [17] and thermal radiometers [18] can be attached to such a goniometer. A difficulty with most of the goniometric setups today is that they are non-automated and their operation is tedious and time-consuming. This causes a lot of problems when measuring thermal directional signatures.

Thermal characteristics of vegetation are influenced by dynamic effects [5]. These dynamic effects consist of changing environmental parameters, like light intensity, sun angle and wind speed [19]. Underlying processes change with these temperatures. As the surface temperature is one of the dominant parameters in the land surface dynamics [20] the change of the underlying processes will directly impact the accuracy of the measurement of the temperature. In order to reduce the dynamic effects during measurements, the directional thermal measurements need to be acquired in a short time span. To understand the diurnal behaviour of the land surface thermal dynamic processes, directional thermal measurements need to be acquired at a frequent, e.g. hourly interval for at least a whole day.

The system presented in this paper enables the researcher to make a complete directional scan in a short time span with high repeat frequency. This was achieved by automating the system and the sensors. The goniometric system was able to complete a directional scan in 5 minutes. Results of optical and thermal directional measurements during the fieldcampaigns of SEN2FLEX 2005, EAGLE 2006, and AGRISAR 2006 will be shown.

Section 2 presents the technical details of the goniometric setup, and Section 3 the results of the field experiments. At the end of Section 3, the limitations of the instrument are discussed, and suggestions for future improvements are given. In Section 4 we conclude this manuscript.

## 2. Materials and Methods

### 2.1. Original Goniometric Setup

A goniometer consists of a rotating arm on which sensors can be mounted. Some of the goniometers used in the field can only change their zenith viewing angle [22] while other goniometers also can set their azimuth angle to an arbitrary value. The additional dimension of rotation is either obtained by a moving train [16–23], or by a boom rotating along a fixed elevated point [24].

The advantage of a goniometer that can only change its zenith viewing angle is that the construction does not need to be very robust and heavy. The disadvantage is that the user has to manually move the system if one needs complete hemispherical coverage. The advantage of a hemispherical system is therefore obvious, although the extra train/boom can make these systems rather heavy.

**Figure 1.**
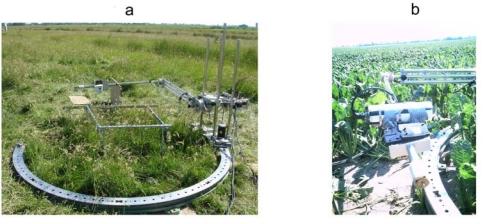
Goniometer in the field. (a) Figure A shows the goniometer in the grassland (tall) during the EAGLE2006 fieldcampaign, Cabauw (The Netherlands). The goniometer, the Irisys thermal camera and the Everest radiometer are shown. (b) Shows the goniometer in the sugar beet field during the AGRISAR2006 fieldcampaign, Demmin (Germany). The CIMEL312-1 radiometer is shown.

**Figure 2.**
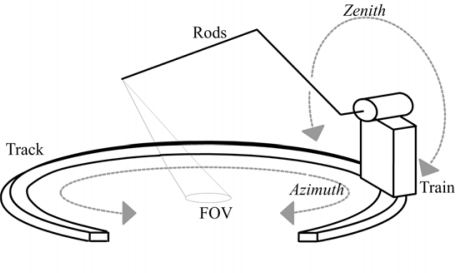
Schematic of the goniometer.

The goniometer used has the same layout as described in [16] (Figures 1 and 2). This system is one of the smallest goniometer available and therefore very mobile. As a result, several field sites can be measured at a high frequency, while retaining the option to easily sample a complete hemisphere. The goniometer consists of two parts: (1) a set of rotating rods connected and (2) a train that runs on a circular track. The rods are connected to the train, and are rotated by motor. The system of rotating rods controls the zenith angle whereas the train controls the azimuth angle. The rail forms two-third of a circle. The 120° gap was purposely left out to reduce the weight of the goniometer, see Figure 6.

### 2.2. Improvements

The original goniometer did not have the option of an automated running mode. For each orientation, zenith and azimuth angles had to be measured manually as the instrument did not have a method of calculating the zenith/azimuth viewing angle operationally. The improvements made to the original goniometric setup included the controlling of the goniometer by laptop and the operational calibration of the motor positions to the viewing angles.

#### 2.2.1. Automated Control

The goniometer is controlled by a control box with sufficient memory to upload a measuring program. This control box only accepts machine code as input. A Matlab program was written to control the positioning of the goniometric system and translate these commands of the movement into machine code.

**Figure 3.**
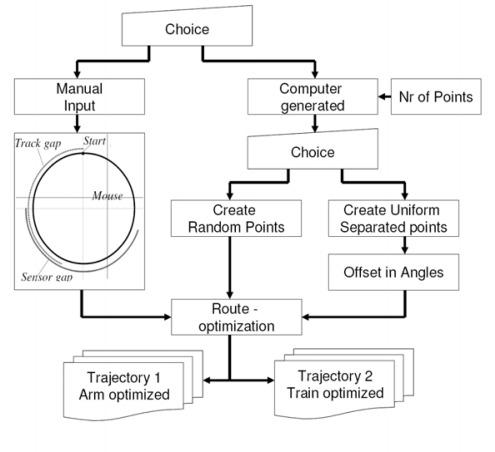
Flowchart of the Matlab Route-calculation program.

The program allows the user to choose between selecting manually the viewing angles or selecting computer-generated viewing angles, see Figure 3. Manually selecting the viewing angles is performed by choosing the orientations on a top-down representation of the goniometer. Selecting the viewing angles by computer-generation can be done for random or uniformly separated angles. In the computer-generated method a predefined offset can be implemented, which could either be solar angles, or north orientation.

The program calculates two trajectories for the goniometer. The first trajectory is arm optimized, the second trajectory is train optimized. As the rotation of the arm is managed by the slowest and weakest motor the arm-optimized trajectory is recommended.

At the selected view angle a delay (user defined duration) is implemented to give the sensors a time to reach an equilibrium state. At the same time a feedback trigger is given to the computer. This trigger can be used for image acquisition start, or to store a timestamp.

#### 2.2.2. Viewing Angles

**Figure 4.**
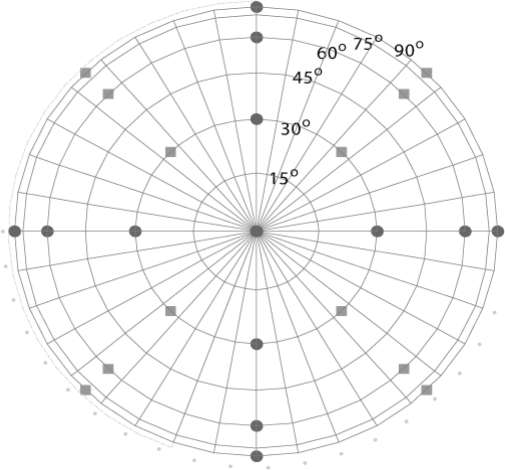
Selected viewing angles as seen from top view of the goniometer. The squares denote the first run and the circles denote the second run.

When performing thermal measurements a short acquisition time is critical. In order to reduce the duration of a single measurement, 25 view angles were chosen, Figure 4. The measurement had to be split into two runs the amount of measuring points to be programmed is limited due to the limited memory capacity of the control box. The two runs were programmed with an offset of 45^°^. The start and end position and the nadir viewing angle were used to compare the change in temperatures.

#### 2.2.3. Operational calibration

The track forms two third of a circle to reduce the weight of the goniometer, leaving a gap of 120 degrees of rails. In this study this gap is used to track the position of the train and thus to calibrate the azimuth angle of the measurement. This is performed by running the train from end to end, and recording the amount of steps the train had to perform. This can be performed at each run.

### 2.3. Sensors

A variety of sensors has been placed on the goniometer. These sensors varied from multi-spectral instruments (CIMEL [18]) to thermal cameras (Irisys 1010 [21–22]). The complete set of thermal instruments used on the goniometer is listed in Table 1. In addition to the thermal instruments, a Canon Digital Camera was placed on the goniometer. The only restriction to the sensor placed on the goniometer is its weight, as the motor controlling the arm-rotation was not strong.

Concurrently to the instruments on the goniometer, other sensors have to be placed in the vicinity of the goniometer. These instruments are needed to investigate the changing environmental conditions. The sensors that were used are listed in Table 2. These sensors measured at 10 Hz interval and the measurements were averaged over 1 minute.

### 2.4. Fieldsites

**Figure 5.**
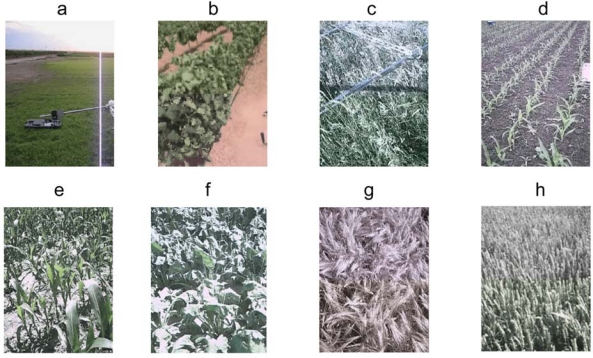
Crops investigated with goniomtric setup. The measurements in Figure A-B are taken in the framework of SEN2FLEX 2005. **(**a) Figure A shows the grassfield (low), (b) B shows a vineyard, (c) C shows grassfield (tall), (d) D shows maize(young), (e) E shows maize(mature), (f) F shows sugarbeet, G shows barley and G shows wheat. The measurements in Figure C-D were taken in the framework of EAGLE 2006. The measurements in Figure E–H were taken in the framework of AGRISAR 2006.

Three datasets were created during three fieldcampaigns: SEN2FLEX2005, EAGLE 2006 and AGRISAR 2006. These fieldcampaigns were performed in Barrax (Spain [22]), Cabauw (the Netherlands [25–26]) and Demmin (Germany [26]). During these fieldcampaigns optical and thermal radiative measurements were performed over variety of crops, see Figure 5.

During the SEN2FLEX2005 campaign measurements over short grass and vineyard were performed. In total 24 runs were performed at day time with an average sampling time of 1 hour. During the EAGLE2006 campaign measurements over tall grassland and young maize were performed. In total 29 runs were performed at day time with an average sampling time of 45 minutes. During the AGRISAR2006 campaign measurements over mature maize, wheat, barley and sugar beet were performed. The total amount of measurements was 15, with only a few runs per crop. The measuring protocol during the SEN2FLEX campaign consisted of only a single run.

## 3. Results and Discussion

A large number of measurements were performed. The results focus on the within species variations and the interspecies differences. In paragraph 3.1 species are compared at different growth stages (tall grass vs. short cut grass and mature maize vs. young maize). In paragraph 3.2 different species are compared to each other. In paragraph 3.3 the succes of the results is discussed.

### 3.1. Within-species Differences in Canopy Structure

#### 3.1.1. Grass

**Figure 6.**
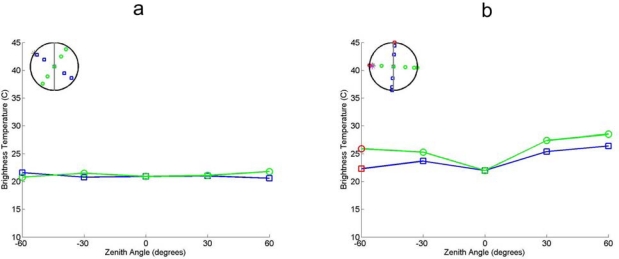
(a). In Figure A the directional brightness temperature of low grass is shown (measurements at 20:43 during the SEN2FLEX campaign), acquired by Everest Radiometer. (b). In Figure B the directional brightness temperature of high grass is shown (measurements at 17:35 during the EAGLE campaign), acquired by Everest Radiometer.

The grassland phenological stages of interest are: short cut (SEN2FLEX) and tall grasses (EAGLE). The brightness temperatures of these stages are shown in Figure 6.

The directional variations are low in both low and high grassland. This is in agreement with expectations; both crops are dense and homogeneous in nature. In high grassland the nadir-looking brightness temperature is a slightly lower compared to other viewing angles. The explanation is that at nadir, the viewing angle is parallel to the grass leaves. The effective cross-section of the grass is therefore the lowest. The contribution of the soil to the observed radiation is therefore the highest. As the soil temperature at the time of acquisition is lower than the vegetation because the sun has at a low zenith angle the emitted radiation of the complete surface is lower at nadir. The low variation in brightness temperature is in agreement with contact temperature measurements taken at the same time of acquisition (not shown in this paper).

#### 3.1.2. Maize

**Figure 7.**
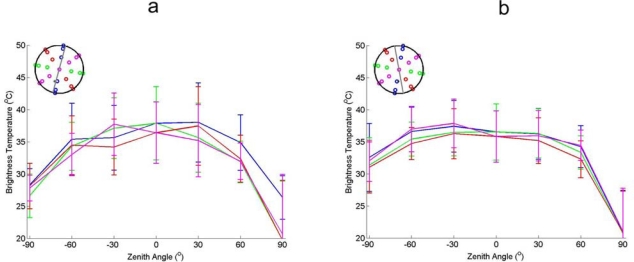
(a). In Figure A, the directional thermal brightness temperatures of young maize is shown (measurements at 12:47 during the EAGLE campaign), acquired by Irisys thermal camera. The error bars denote the standard deviation of the brightness temperatures in the image to the mean brightness temperatures. (b). In Figure B, the directional thermal brightness temperatures of mature maize is shown (measurements at 13:01 during the AGRISAR campaign), acquired by Irisys thermal camera.

The Maize phenological stages of interest are defined as: young (EAGLE) and mature (AGRISAR). The brightness temperatures of these stages are shown in Figure 7.

The directional variations in the brightness temperature are present in both young and mature maize. The directional behaviour for young maize is only pronounced at the very low viewing angles (−90^°^ and +90^°^). At very low angles the individual maize plants appear to form a closed canopy, whereas at normal viewing angles the individual maize plants do not form a closed canopy. As the soil temperature during time of acquisition was higher than the canopy temperature the brightness temperature is higher when observing more soil. Variations in measured contact temperature (not shown in this paper) of the different components agree with the above explanation.

### 3.2. Inter-species Differences in Canopy Structure

**Figure 8.**
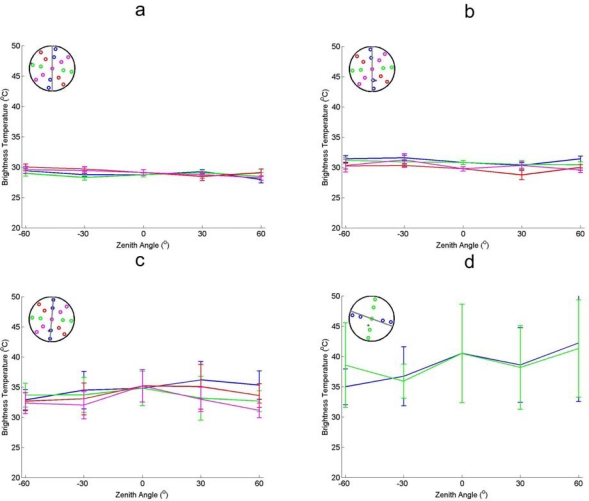
Thermal brightness temperatures obtained by Irisys thermal camera over different crops. (a) Figure A shows barley at 18:08 (AGRISAR) (b). B shows wheat at 11:30 (AGRISAR), (c) C shows sugar beet at 12:43 (AGRISAR) (d). D shows vineyard at 16:30 (SEN2FLEX). The error bars denote the standard deviation of the brightness temperatures in the image to the mean brightness temperatures.

A comparison of the directional brightness temperature of the different canopies is performed. Figure 8 shows the results for barley, wheat, sugar beet and vineyard. The canopies are presented in order of structural complexity.

Barley and wheat display the same directional thermal behaviour as the high grassland, with very low directional behaviour. Similar to long grass canopies of barley and wheat consist of long stems and short leaves. The only difference between the grassland and the two crops in Figure 3 is the mean temperature. This is caused by the higher canopy temperature at time of acquisition during the AGRISAR campaign and the EAGLE campaign.

Sugar beet displays more directional thermal behaviour than the barley and wheat, but less than maize (Figure 7). This is caused by the severe drought that occurred during the AGRISAR campaign, causing wide leaves to rest horizontally on the ground. The horizontal orientation of the leaves and the coverage of the soil reduce the directional thermal signature.

The Vineyard displays large but inconsistent directional variations in the thermal emission. The reason is that the spatial scale of variations in the crop are larger compared to the field of view of the sensor. As a result the ratio of soil/canopy in the image is not constant. This is for the most part caused by the variations in soil-canopy ratio and will be discussed further in the next paragraph.

### 3.3. Discussion

**Figure 9.**
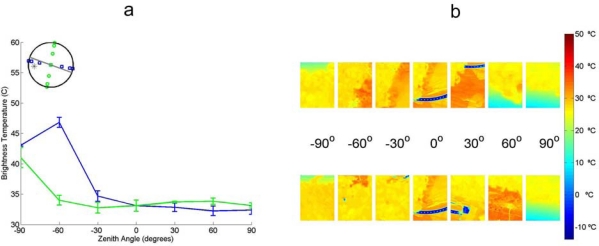
Comparison of CIMEL and Thermotracer acquired brightness temperatures. (a). In Figure A the directional brightness temperatures acquired at 16:30 by the CIMEL 312-1 (band 1: 8–14μm) is shown. The error bars denote the difference of band 1 to the other bands. (b). In Figure B the brightness temperatures at different angles by is shown; acquired by Thermotracer at acquired at 18:89.

The variations observed in Figure 8d are not shown in the CIMEL 312 measurements, shown in Figure 9a. The difference originates from the statistical methods (average, standard deviation) used to generate Figure 8d. As can be seen from Figure 9b the ratio of canopy to soil is not constant for different view angles. The aggregation of the soil emitted and canopy emitted thermal radiation causes this average brightness temperature to give strange results. As the view angle of the CIMEL camera was adjusted per acquisition point to have the same ratio of vegetation to soil.

The kinematic temperature of the different components can still be identified [21]. However this method is very hard to upscale to satellite borne sensors. Therefore a larger goniometer has to be used to measure correctly over the vineyard. This goniometer will then be able to capture multiple rows at the same time. The ratio of soil vs. vegetation will then remain constant.

## 4. Conclusions

This paper presents a technique to obtain fast directional measurement with a goniometer. These fast measurements are possible by automating the goniometric system and the attached sensors. The goniometer is able to complete a run within four minutes. This acquisition time is within the timeframe normally posed for kinematic temperature changes in canopies. Therefore this goniometer is suited for making thermal directional measurements.

The results of the thermal directional measurements over different crops have been shown. These crops are: short and tall grassland, young and mature maize, barley, wheat, sugar beet and vineyard. Of all these crops the grassland had the lowest structural complexity and the vineyard the highest structural complexity. The directional radiative thermal signatures corresponded very well to expectations for all but the vineyard.

Due to the size of the vineyard compared to the size of the goniometer the soil–canopy ratio was not constant. This resulted in unexpected directional signatures. The results can be used to retrieve the component brightness temperatures of soil and leaves.

## Figures and Tables

**Table 1.** Thermal instruments used on the goniometer.

	**Accuracy (K)**	**Spectral Res. (K)**	**IFOV^°^**	**Specifics**

**Thermal Radiometers**				

CIMEL 312-1	±0.5	0.05	10	4 Bands
CIMEL 312-2	±0.5	0.05	10	6 Bands
Everest 3000	±0.5	0.10	20	broadband

**Thermal Cameras**				

Thermal Irisys 1010	±0.5	0.10	20.0 × 20.0	16 × 16 pixels
Thermotracer TH9100 Pro	±2.0	0.06	21.7 × 16.4	320 × 240 pixels

**Table 2.** Supplemental instruments used during the directional acquisition of thermal radiation.

Net radiation Optical	Campbell CNR1
Net radiation Thermal	Campbell CNR1
Wind speed	Campbell A100R
Wind direction	Campbell W200P
Pressure	Campbell PTB101B
Humidity	Campbell HMP45C
Air temperature	Campbell HMP45C
Soil temperature	Campbell 107T
Kinematic temperatures	SEMI 833 ET
